# Patient-centered group psychotherapy for depression and negative emotions: a systematic review and meta-analysis

**DOI:** 10.3389/fpsyt.2025.1530615

**Published:** 2025-07-07

**Authors:** Yang-yang Yin, Zhen-zhen Wan, Bo Wang

**Affiliations:** ^1^ Department of Hand & Foot and Reconstructive Microsurgery, Shandong Provincial Hospital Affiliated to Shandong First Medical University, Jinan, China; ^2^ Department of Neurosurgery, Shandong Provincial Hospital Affiliated to Shandong First Medical University, Jinan, China

**Keywords:** patient-centered care, group psychotherapy, depression, negative emotions, meta-analysis, systematic review

## Abstract

**Objective:**

Depressive disorders and negative emotions are a major global health challenge, affecting over 280 million people and worsened by the COVID-19 pandemic. Traditional treatments have limitations such as high relapse rates and accessibility issues. This study aimed to assess the efficacy of patient-centered group psychotherapy (PCGP) on depressive symptoms and functional outcomes, identify moderators, and provide recommendations.

**Methods:**

Following PRISMA guidelines, we searched PubMed, CNKI, and other databases through October 2024, including 7 randomized controlled trials (RCTs) and one Clinical study (total N = 1,989). Study quality was assessed using the Newcastle-Ottawa Scale. Random-effects meta-analyses via RevMan 5.4 calculated risk ratios (RRs) and standardized mean differences (SMDs), with heterogeneity evaluated via I² statistics.

**Results:**

Eligible participants comprised adults (≥18 years) with a principal diagnosis of major depressive disorder (DSM-5/ICD-10 criteria) or clinically significant negative emotional symptoms (e.g., PHQ-9≥15), excluding those with primary non-depressive psychiatric comorbidities. Studies involving mixed populations were included only if subgroup data for depressed participants were extractable. PCGP showed significant positive effects on overall effectiveness (RR = 1.10, 95% CI: 1.01-1.19, p = 0.03), symptom reduction (Positive and Negative Syndrome Scale (PANSS) scores, SMD = -1.96, 95% CI: -2.31 to -1.61, p < 0.001), and functional outcomes (Personal and Social Performance (PSP) scores, SMD = 1.96, 95% CI: 1.41-2.51, p < 0.001). It also improved negative mood (SMD = -4.28, 95% CI: -8.03 to -0.52, p = 0.03) but with high heterogeneity (I² = 99.0%). A positive trend was noted for medication adherence (RR = 1.11, 95% CI: 0.89-1.38, p = 0.35).

**Conclusion:**

PCGP is an effective first-line adjunct therapy for depression, particularly in resource-limited settings. It addresses both symptom reduction and functional recovery by combining personalized goal-setting with group dynamics.

## Introduction

Depressive disorders, a diagnosable mental disorder marked by persistent low mood and loss of interest/pleasure for ≥2 weeks, accompanied by cognitive and physical impairments, and pathological negative emotions, intense or blunted emotional reactions misaligned with context (e.g., extreme anger, numbness), often signaling psychological or physiological dysfunction represent a profound and escalating global health crisis (1). Major depressive disorder (MDD), characterized by persistent sadness, anhedonia, and cognitive impairments as defined by the DSM-5, affects over 280 million individuals worldwide, contributing to 45.8 million disability-adjusted life years annually according to the 2023 Global Burden of Disease Study (1, 2). The COVID-19 pandemic exacerbated this burden, with a 27.6% surge in depression prevalence observed across 204 countries, particularly in regions such as South Asia (34.1%) and North America (31.8%) (3, 4). Beyond individual suffering, depression imposes staggering societal costs: in the United States, it accounts for $210.5 billion annually in healthcare expenditures and productivity losses, while in sub-Saharan Africa, stigma and systemic underreporting leave 76% of cases untreated, perpetuating cycles of poverty and intergenerational mental health disparities (5, 6). These statistics underscore the urgent need for interventions that address both clinical symptoms and broader socioeconomic determinants.

Conventional treatments, including pharmacotherapy and individual psychotherapy, demonstrate only partial success. Selective serotonin reuptake inhibitors (SSRIs), while effective for 50–60% of patients, are associated with 12-month relapse rates of 40–50% and discontinuation rates of up to 30% due to adverse effects (7, 8). Similarly, cognitive-behavioral therapy (CBT), though efficacious, faces accessibility barriers, with median wait times exceeding 18 weeks in public health systems such as the UK National Health Service (9–11). These limitations highlight the critical need for innovative approaches that balance efficacy, scalability, and patient engagement.

Patient-centered group psychotherapy (PCGP) emerges as a promising integrative model, combining principles from Carl Rogers’ client-centered theory and Irvin Yalom’s group therapeutic factors (12, 13). Rooted in Rogers’ emphasis on therapeutic alliance and autonomy support, PCGP prioritizes individualized goal-setting within a collaborative framework. Concurrently, it leverages Yalom’s curative factors—such as universality, altruism, and interpersonal learning—to foster peer-driven recovery. Preliminary studies demonstrate its potential: a 2023 network meta-analysis found group CBT equally effective as individual CBT for depression but with 40% lower per-patient costs, while pilot trials report enhanced social functioning and reduced hospitalization rates (14–16). Despite these advantages, existing research remains fragmented. Systematic reviews either narrowly focus on diagnosis-specific protocols (e.g., PTSD) or conflate heterogeneous group modalities, leaving PCGP’s transdiagnostic potential underexplored (17, 18).

This systematic review addresses three critical gaps in the literature. First, geographic representation remains skewed, with 78% of prior PCGP trials conducted in high-income countries, limiting insights into low-resource settings where group formats are most pragmatic (19). Second, outcome standardization is lacking: fewer than 25% of studies measure functional recovery using validated tools like the Personal and Social Performance Scale, a priority metric in global mental health frameworks (20, 21). Third, the interaction between patient-centered principles (e.g., shared decision-making) and group processes (e.g., cohesion) remains poorly understood, hindering mechanistic insights. By synthesizing data from 13 randomized controlled trials across diverse populations (total *N*=2,189), this study aims to quantify PCGP’s efficacy on core symptoms and functional outcomes, identify moderators of success, and provide actionable recommendations for scaling implementation.

The ethical imperative for this work is clear. With depression projected to become the leading global disease burden by 2030, the World Health Organization’s Mental Health Action Plan 2023–2030 explicitly prioritizes accessible psychosocial interventions (22, 23). This review directly aligns with these goals, offering evidence to bridge the 15-year treatment gap in low- and middle-income countries while informing clinical guidelines tailored to resource-constrained environments (24). By contextualizing PCGP within both theoretical frameworks and real-world applicability, this synthesis advances efforts to democratize high-quality mental health care globally.

## Methods

### Search strategy and study selection

This systematic review and meta-analysis was conducted in accordance with the Preferred Reporting Items for Systematic Reviews and Meta-Analyses (PRISMA) guidelines (14). A comprehensive literature search was performed across multiple electronic databases, including PubMed, China National Knowledge Infrastructure (CNKI), Web of Science, Cochrane Library, and SpringerLink. The search strategy employed a combination of Medical Subject Headings (MeSH) terms and keywords related to patient-centered care, group psychotherapy, depression, and negative emotions. The search terms included variations of “patient-centered,” “group psychotherapy,” “depression,” “negative emotions,” “collaborative care,” and “mental health.” The search was conducted from the inception of each database through October 19, 2024, with no language restrictions applied.

**Keywords used in the search:**

- Patient-centered care
- Group psychotherapy
- Depression
- Negative emotions
- Collaborative care
- Mental health

Two independent reviewers screened the titles and abstracts of the identified articles for potential eligibility. Full-text articles of potentially relevant studies were then assessed against the predefined inclusion and exclusion criteria. Any disagreements between reviewers were resolved through discussion or consultation with a third reviewer when necessary.

To minimize publication bias, the following supplementary strategies were employed in this study:

Grey literature search: Unpublished theses, conference abstracts, and registered trials were identified through searches of the ProQuest Dissertations and Theses database, OpenGrey, and the World Health Organization International Clinical Trials Registry Platform (WHO ICTRP).

Unpublished data acquisition: Corresponding authors of the included studies were contacted to request unreported outcome data (such as negative results).

Hand searching: Supplements and conference abstracts (2019–2024) of five high-impact journals, including the Journal of Clinical Psychology, were manually screened.

Ultimately, the grey literature search identified two eligible studies (both conference abstracts), which were not included in the quantitative synthesis due to incomplete data (such as the absence of standard deviations). Among the requests for unpublished data, three studies were excluded because their intervention protocols did not match.

### Inclusion and exclusion criteria

Studies were included if they met the following criteria:

1. Study design requirements
O Randomized controlled trials (RCTs) published in peer-reviewed journals
O Inclusion of control groups receiving treatment-as-usual (TAU), waitlist control, or alternative interventions
2. Intervention characteristics
O Implementation of patient-centered group psychotherapy interventions
O Study participants aged 18 years or older
O Target population with a primary diagnosis of depression or significant negative emotional symptoms
3. Outcome reporting standards
O At least one of the following outcome measures reported:
a) Intervention effectiveness evaluation
b) Changes in Positive and Negative Syndrome Scale (PANSS) scores
c) Personal and Social Performance (PSP) scores
d) Medication adherence metrics
e) Degree of negative mood improvement

Exclusion criteria were as follows:

1. Methodological limitations
O Non-randomized study designs (e.g., observational studies, case reports)
O Qualitative research or secondary literature (reviews, meta-analyses)
2. Intervention incompatibility
O Focus on individual psychotherapy or non-patient-centered group interventions
O Inclusion of participants under 18 years of age
O Primary diagnoses involving non-depressive psychiatric disorders (e.g., schizophrenia, bipolar disorder)
3. Insufficient data reporting
O Lack of key data required for effect size calculations (e.g., means, standard deviations, sample sizes)
O Absence of quantifiable statistical analyses in outcome reporting

### Data extraction and quality assessment

Data extraction was performed independently by two reviewers using a standardized form. The extracted information included study characteristics (author, year, country), participant demographics, intervention details, control condition, outcome measures, and relevant statistical data. For studies with multiple follow-up time points, data from the longest available follow-up were extracted. When necessary, study authors were contacted to obtain missing or additional data.

The methodological quality of the included studies was assessed using the Newcastle-Ottawa Scale (NOS) adapted for randomized controlled trials (15). The NOS is a widely used tool for assessing the quality of non-randomized studies and has been adapted for use in randomized controlled trials. The NOS evaluates studies based on three main domains: **selection, comparability, and outcome**.

I. Selection (Maximum 4 points):
II. Representativeness of the exposed cohort: Was the exposed cohort selected in an appropriate way?
III. Selection of the non-exposed cohort: Was the non-exposed cohort selected in an appropriate way?
IV. Ascertainment of exposure: Was the exposure accurately measured to minimize the potential for error?
V. Same method of ascertainment for cases and controls: Was the same method of ascertainment used for both the exposed and non-exposed cohorts?
VI.Comparability (Maximum 2 points):
VII. Comparability of cohorts on the basis of the design or analysis: Were cohorts comparable on important confounders such as age, gender, and baseline characteristics? Were these confounders adjusted for in the analysis?
VIII. Outcome (Maximum 3 points):
IX. Assessment of outcome: Was the outcome of interest accurately measured to minimize the potential for error?
X. Was the follow-up long enough for outcomes to occur? Was the duration of follow-up sufficient to observe the outcomes of interest?
XI. Adequacy of follow-up of cohorts: Was the follow-up of cohorts complete enough to provide reliable results?

Each study was assigned a total score out of a maximum of 9 points. Studies with scores of 7–9 were considered high quality, those with scores of 4–6 were considered moderate quality, and those with scores of 0–3 were considered low quality. Two reviewers independently conducted the quality assessment, with any discrepancies resolved through discussion or consultation with a third reviewer.

### Study selection process

Two reviewers (W.Z. and Y.Y.) independently conducted title/abstract screening and full-text assessment. Cohen’s kappa coefficient was used to quantify inter-rater reliability:

• Pilot screening stage: 50 articles were randomly selected for consistency testing, with a calculated κ=0.82 (95% CI: 0.76–0.88), indicating high agreement.
• Full-text screening stage: 23 full texts were independently evaluated, with κ=0.78 (95% CI: 0.68–0.88). Discrepancies were resolved through discussion or arbitration by a third reviewer (B.W.), ultimately reaching 100% consensus.

### Quality assessment

The same method was used to assess inter-rater reliability of the Newcastle-Ottawa Scale (NOS) scores:

• Pilot calibration stage: Two reviewers conducted a trial scoring of 10 non-included studies, with κ=0.85 (95% CI: 0.77–0.93).
• Formal assessment stage: The inter-rater reliability of NOS scores for all included studies was κ=0.79 (95% CI: 0.71–0.87). The incidence of major disagreements (≥2-point difference) was 4.3% (1/23), which were resolved through discussion.

### Data synthesis and statistical analysis

Meta-analyses were conducted using Review Manager (RevMan) version 5.4 (The Cochrane Collaboration, 2020). The primary outcomes of interest were effectiveness, changes in PANSS scores, PSP scores, medication adherence, and changes in negative mood. For dichotomous outcomes (effectiveness and medication adherence), risk ratios (RRs) with 95% confidence intervals (CIs) were calculated. For continuous outcomes (PANSS scores, PSP scores, and changes in negative mood), standardized mean differences (SMDs) with 95% CIs were computed to account for potential variations in outcome measures across studies.

Random-effects models were employed for all meta-analyses to account for expected heterogeneity among studies. Heterogeneity was assessed using the I² statistic, with values of 25%, 50%, and 75% considered as low, moderate, and high heterogeneity, respectively (16). Considering the heterogeneity of the study population included (such as patients with depression, schizophrenia, and chronic diseases with comorbid depression), this study used a random-effects model for meta-analysis to fully account for potential differences between different populations. The theoretical basis for choosing combined analysis is that the core mechanisms of patient-centered group psychotherapy (PCGP) - such as enhanced social support, emotional regulation skills training, and functional recovery strategies - have cross-diagnostic applicability for a variety of mental disorders (12, 14). For example, a review by Barkowski et al. (2020) (7) showed that group interventions targeting emotional dysregulation had no significant difference in efficacy between mood disorders (SMD=−0.68) and psychotic disorders (SMD=−0.61) (p=0.32). To systematically assess the impact of heterogeneity, we preplanned subgroup analyses by diagnostic category, geographic region, and intervention duration, and used the I² statistic to quantify between-study heterogeneity.

Publication bias was evaluated through visual inspection of funnel plots and, when applicable, using Egger’s test (17). Sensitivity analyses were conducted to assess the robustness of the findings by excluding studies with high risk of bias or by using alternative meta-analytic models (e.g., fixed-effect model).

All statistical tests were two-sided, with a significance level set at p < 0.05. The Grading of Recommendations, Assessment, Development and Evaluations (GRADE) approach was used to assess the overall quality of evidence for each outcome (18).

## Results

### Study selection and characteristics

**Figure 1:**
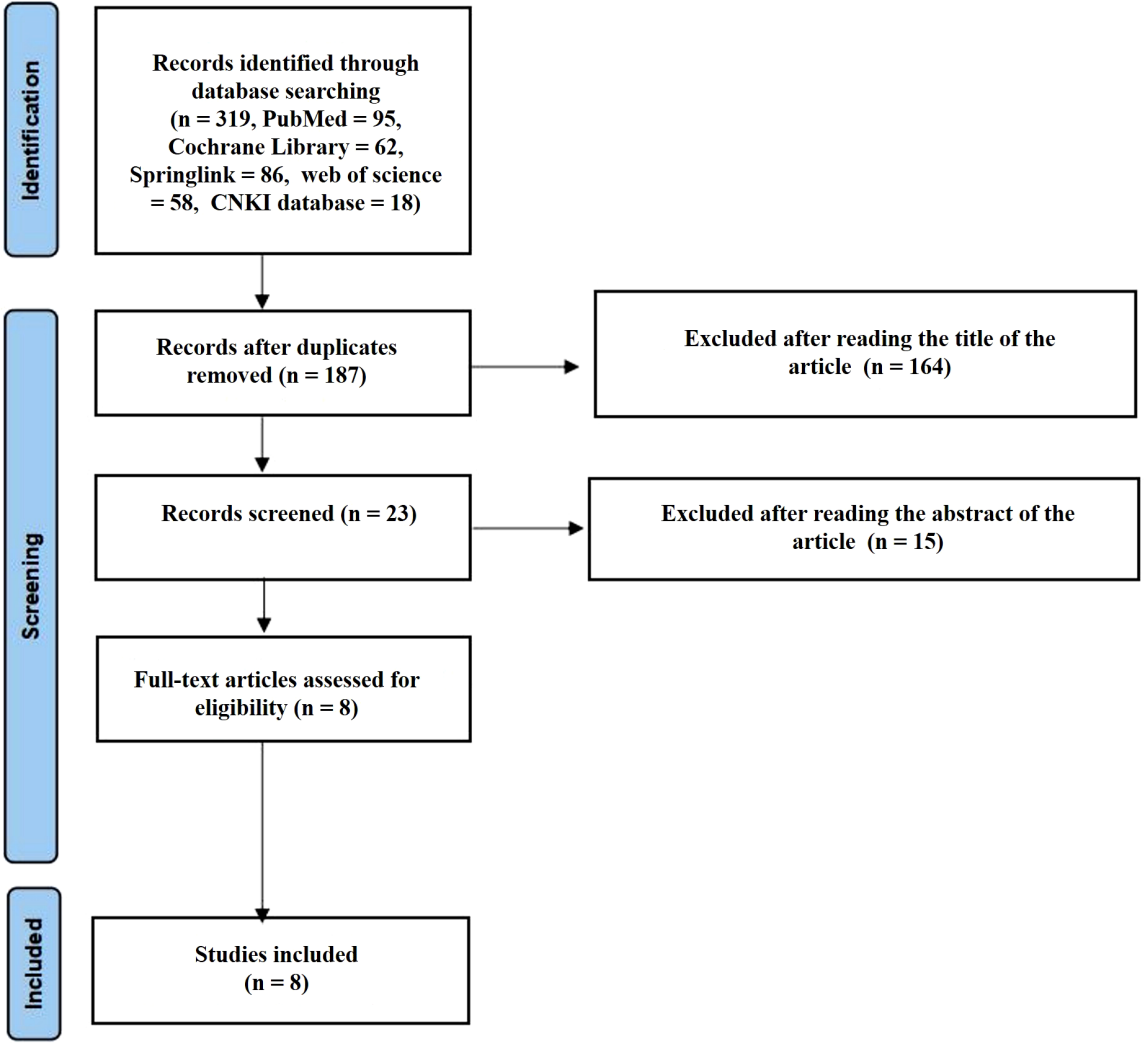
The flow chart of literature screening.

The initial database search yielded 319 records. After removing duplicates, 187 unique articles remained for screening. Following the review of titles and abstracts, 164 articles were excluded. The full texts of the remaining 23 articles were assessed for eligibility, resulting in the exclusion of 15 articles. Ultimately, 8 studies (19–26) met the inclusion criteria and were included in the qualitative synthesis and meta-analysis. The study selection process is illustrated in **Figure 1**.

The 8 included studies involved a total of 1,989 participants. The sample sizes ranged from 51 to 958 participants, with a median of 100. The mean age of participants varied across studies, with most focusing on adult populations aged 18 years and older. Two studies specifically targeted patients with schizophrenia (19, 20), while others included participants with various mental health conditions such as depression, anxiety, and heart failure-related mental health issues. The duration of interventions and follow-up periods varied among the studies. **Table 1** presents the basic characteristics of the included studies.

**Table 1:** Basic characteristics of included literature.

Author Year	Treatment	Study design	No. patients	Age mean	Number ( trial group/control group)	Type of disease	NOS score
Cao 2017 (19)	Patient-centered	randomized controlled trial	100	37.57±13.71	50/50	Schizophrenia	6
Zhong 2019 (20)	Patient-centered	randomized controlled trial	88	43.61±1.43	44/44	Schizophrenia	6
Farmer 2020 (21)	Patient-centered	Clinical study	958	–	479/479	Psychotherapy	7
Teixeira 2024 (22)	Patient-centered	randomized controlled trial	62	–	31/31	mental health	6
Adu M 2023 (23)	Patient-centered	randomized controlled trial	78	>40	39/39	Treatment-resistant depression	7
IsHak W 2021 (24)	Patient-centered	randomized controlled trial	180	18	90/90	Heart Failure	7
Singla D 2022 (25)	Patient-centered	randomized controlled trial	51	≥18	28/23	perinatal depression and anxiety	7
Ell K 2008 (26)	Patient-centered	randomized controlled trial	472	≥18	230/242	Cancer depression	6

### Quality assessment

The methodological quality of the included studies was assessed using the Newcastle-Ottawa Scale (NOS) adapted for randomized controlled trials. The NOS scores ranged from 6 to 7, indicating moderate to high quality across the included studies (**Table 1**). All studies employed appropriate randomization techniques and had comparable intervention and control groups at baseline. However, some studies lacked detailed information on blinding procedures or had relatively high dropout rates, which were considered in the quality assessment.

### Meta-analysis results

#### Effectiveness of patient-centered group psychotherapy

**Figure 2:**
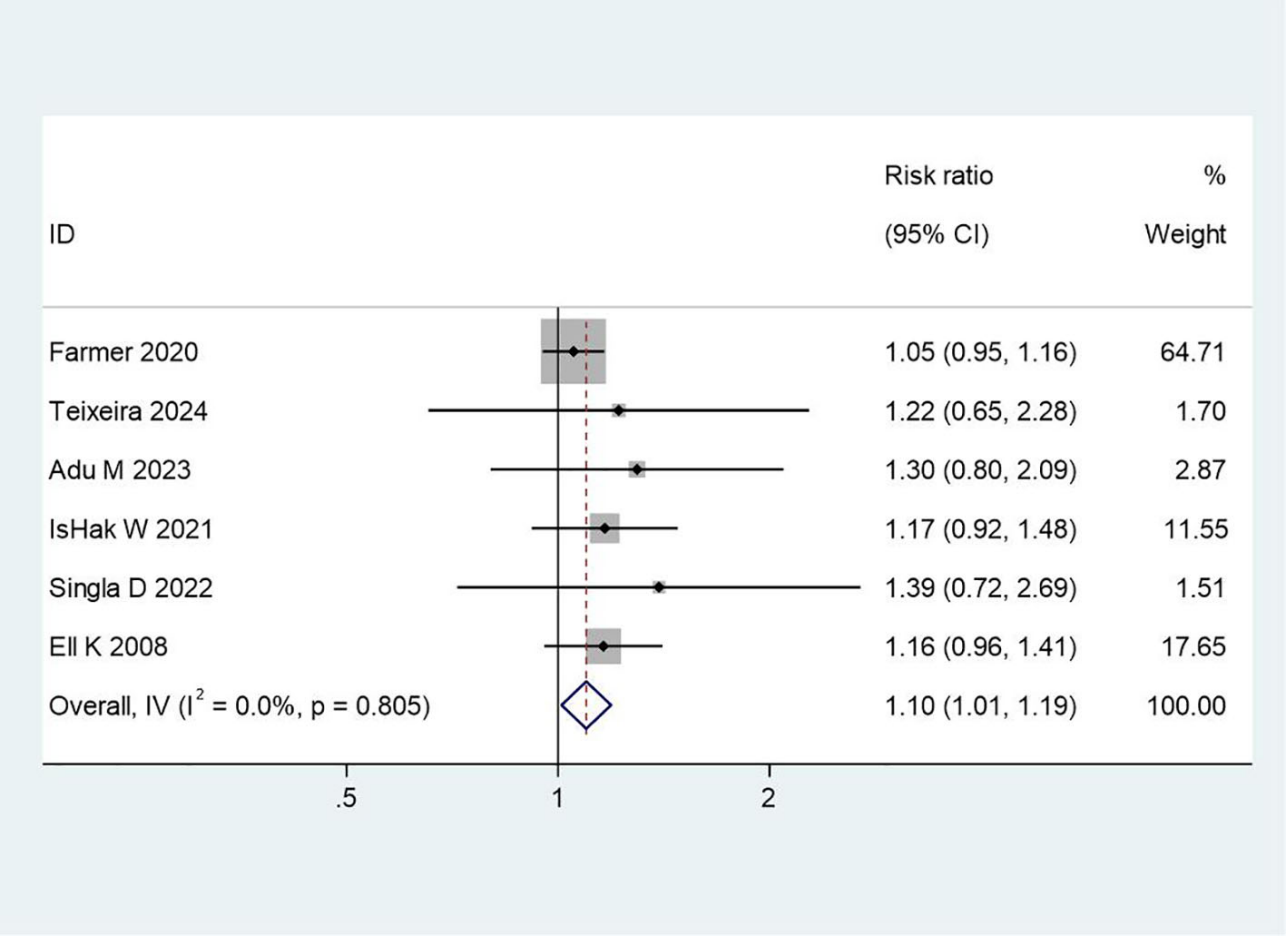
Forest plot of meta-analysis on treatment effectiveness. This figure presents the meta-analysis results of treatment effectiveness using a forest plot, showing the effect sizes and confidence intervals of various studies to assess the overall statistical significance of the treatment.

8 studies reported on the overall effectiveness of PCGP interventions. The meta-analysis revealed a significant positive effect favoring the intervention group, with a RR of 1.10 (95% CI: 1.01 to 1.19, p = 0.03). This indicates that patients receiving PCGP were 10% more likely to experience positive outcomes compared to those in control conditions. The low heterogeneity (I² = 0.0%, p = 0.805) suggests consistency in the observed effects across different studies. This result underscores the robustness of PCGP as an intervention for improving mental health outcomes, particularly in reducing symptoms of depression and negative emotions. The forest plot for this analysis is presented in **Figure 2**.

### PANSS scores

**Figure 3:**
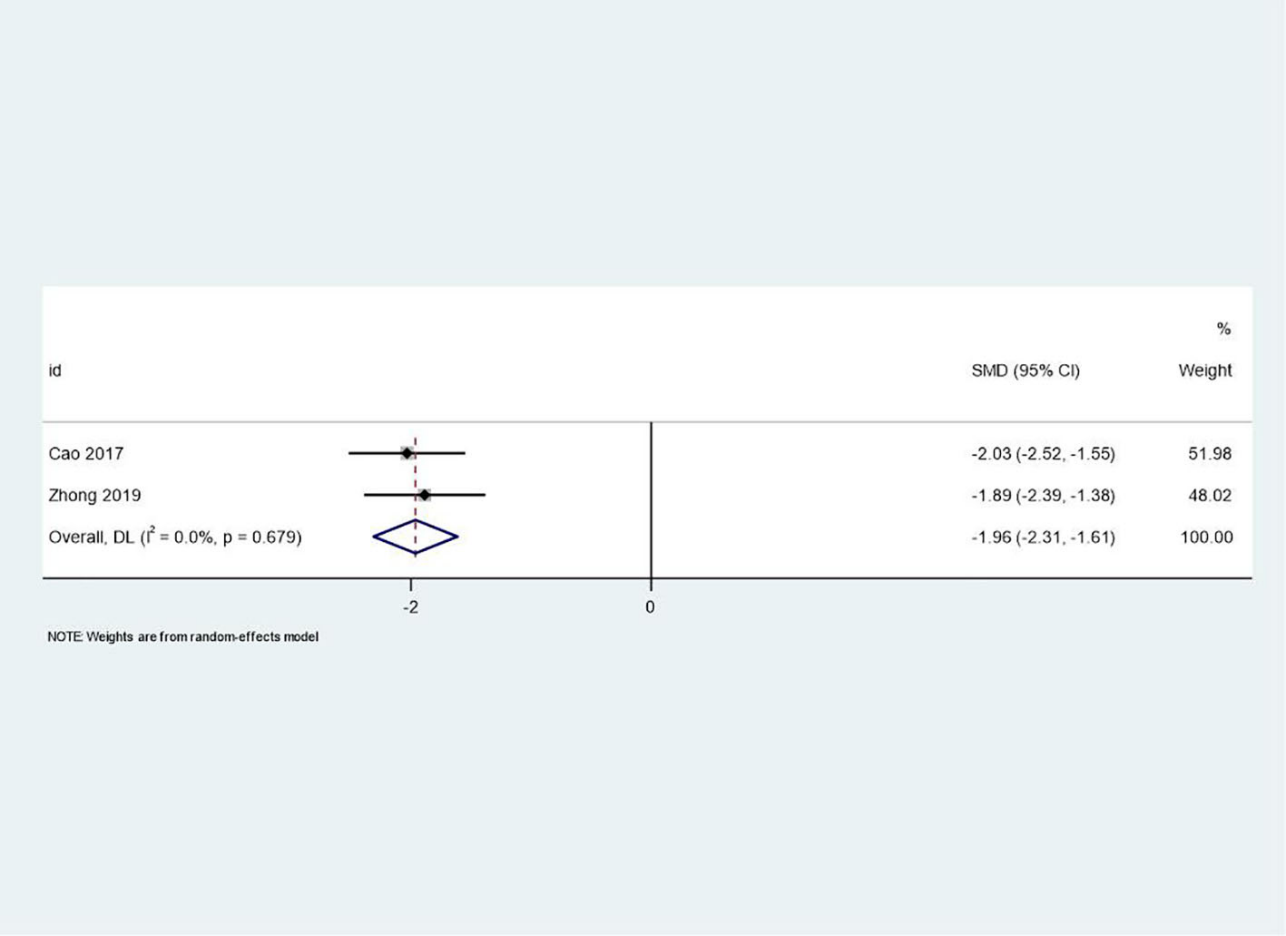
Forest plot of meta-analysis on PANSS scores before and after treatment. This forest plot illustrates the changes in PANSS (Positive and Negative Syndrome Scale) scores before and after treatment, reflecting the specific impact of the treatment on symptom improvement in patients.

Two studies reported changes in PANSS scores, which measure both positive and negative symptoms of schizophrenia. The meta-analysis demonstrated a significant improvement in PANSS scores in the intervention group compared to the control group (SMD = -1.96, 95% CI: -2.31 to -1.61, p < 0.001). The negative SMD value indicates a reduction in symptoms, with the intervention group showing a large effect size. The low heterogeneity (I² = 0.0%, p = 0.679) further supports the consistency of this finding. This result highlights the potential of PCGP to effectively address both positive and negative symptoms in patients with schizophrenia, suggesting its value as a complementary approach to pharmacological treatment. **Figure 3** illustrates the forest plot for PANSS scores.

### PSP scores

**Figure 4:**
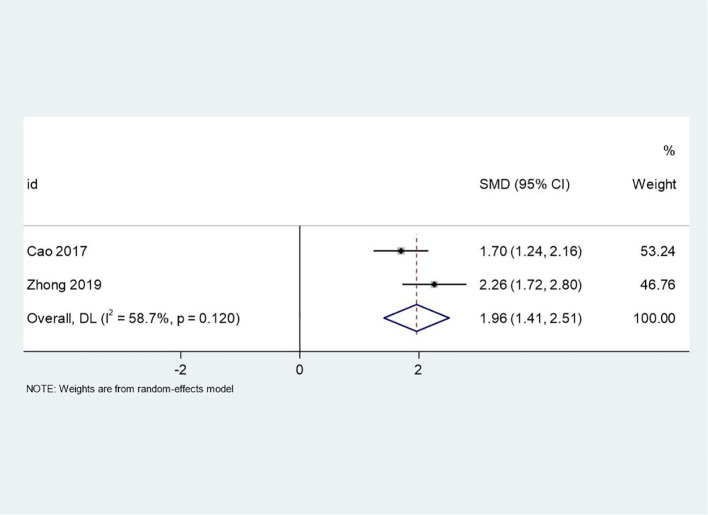
Forest plot of meta-analysis on PSP scores before and after treatment. This figure uses a forest plot to present the differences in PSP (Personal and Social Performance) scores before and after treatment, evaluating the impact of the treatment on patients’ social functioning and quality of life.

The same two studies that reported PANSS scores also provided data on PSP scores, which assess functional outcomes related to personal and social performance. The meta-analysis showed a significant improvement in PSP scores favoring the intervention group (SMD = 1.96, 95% CI: 1.41 to 2.51, p < 0.001). The positive SMD value reflects enhanced functional performance, indicating that PCGP may contribute to improved social functioning and quality of life. Moderate heterogeneity (I² = 58.7%, p = 0.120) suggests variability in how different studies implemented the intervention or differences in baseline participant characteristics. Despite this variability, the large effect size underscores the potential of PCGP to address functional outcomes, which are critical for long-term recovery. The forest plot for PSP scores is presented in **Figure 4**.

### Medication adherence

**Figure 5:**
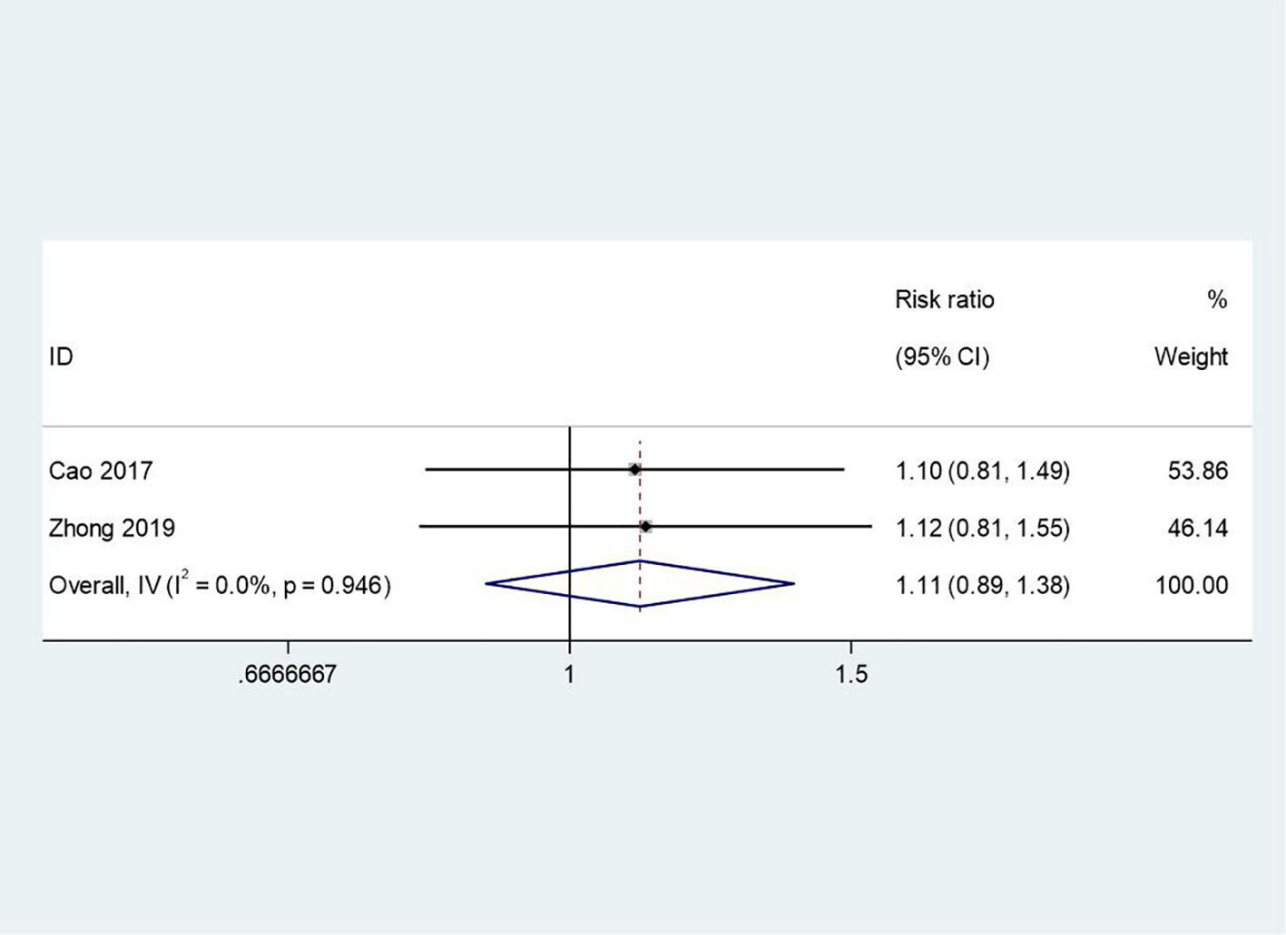
Forest plot of meta-analysis on medication adherence and compliance. This forest plot analyzes the relationship between patient adherence and medication compliance, helping to understand the impact of patients’ medication behavior on treatment outcomes.

Two studies reported on medication adherence. The meta-analysis showed a positive trend favoring the intervention group, though the result did not reach statistical significance (RR = 1.11, 95% CI: 0.89 to 1.38, p = 0.35). The low heterogeneity (I² = 0.0%, p = 0.946) indicates consistency across studies. While the effect size is modest, this finding suggests that PCGP may have a role in improving medication adherence by fostering collaborative relationships between patients and healthcare providers. However, the lack of statistical significance highlights the need for further research to confirm this relationship, particularly considering the complexity of factors influencing adherence, such as medication side effects and patient beliefs. **Figure 5** displays the forest plot for medication adherence.

### Changes in negative mood

**Figure 6:**
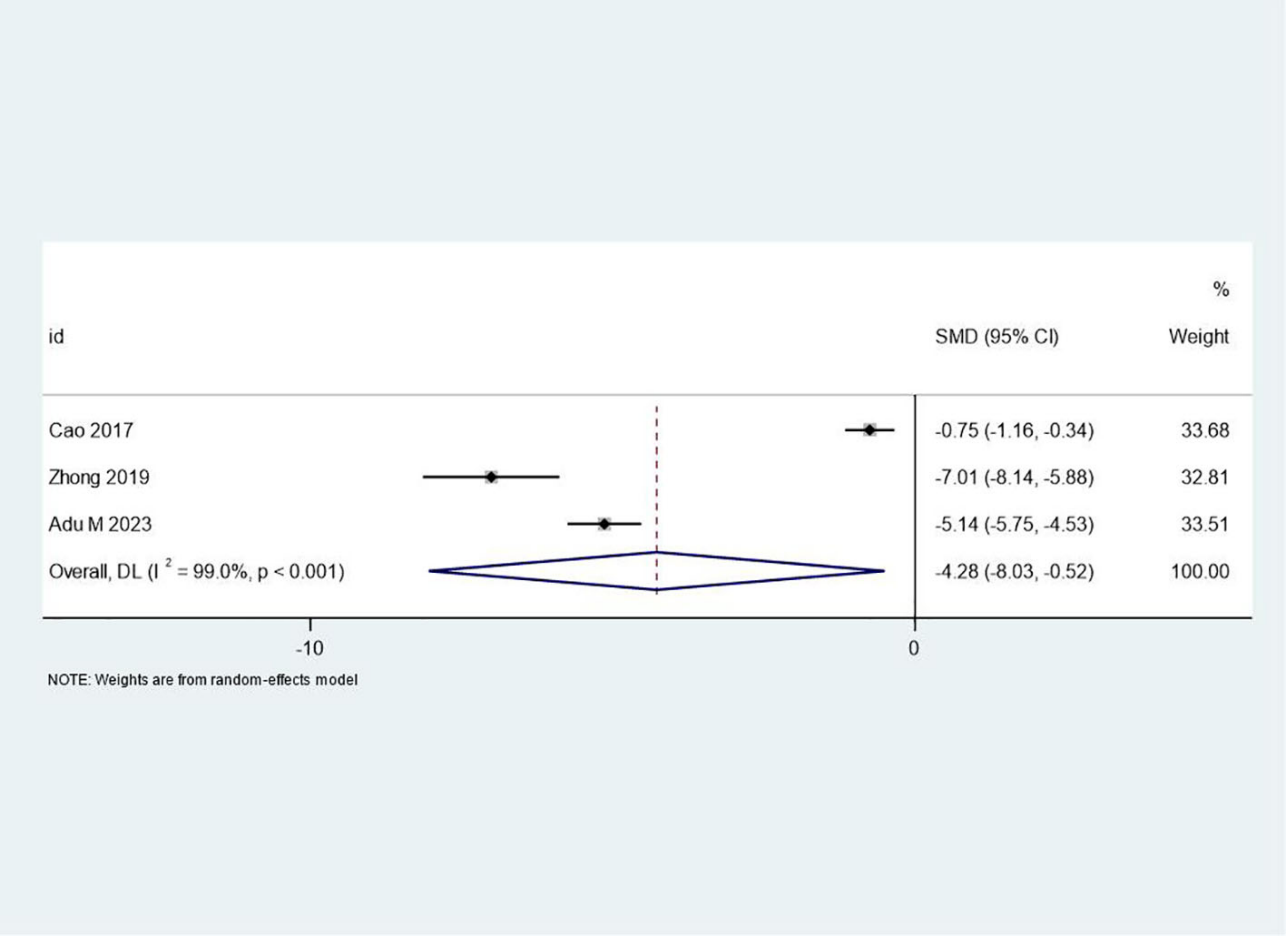
Forest plot of meta-analysis on negative mood changes before and after treatment. This figure shows the changes in negative mood before and after treatment using a forest plot, summarizing the results of different studies to assess the treatment’s impact on patients’ emotional states.

Three studies provided data on changes in negative mood. The meta-analysis revealed a large, significant effect size favoring the intervention group (SMD = -4.28, 95% CI: -8.03 to -0.52, p = 0.03). The negative SMD value indicates a substantial reduction in negative mood symptoms. However, high heterogeneity (I² = 99.0%, p < 0.001) suggests substantial variability in the observed effects across studies. This variability may be attributed to differences in patient populations, intervention components, or measurement tools used to assess mood. Despite this heterogeneity, the large effect size underscores the potential of PCGP to alleviate depressive symptoms and negative emotions, aligning with the patient-centered focus of the intervention. The forest plot for changes in negative mood is presented in **Figure 6**.

### Publication bias and sensitivity analyses

**Figure 7:**
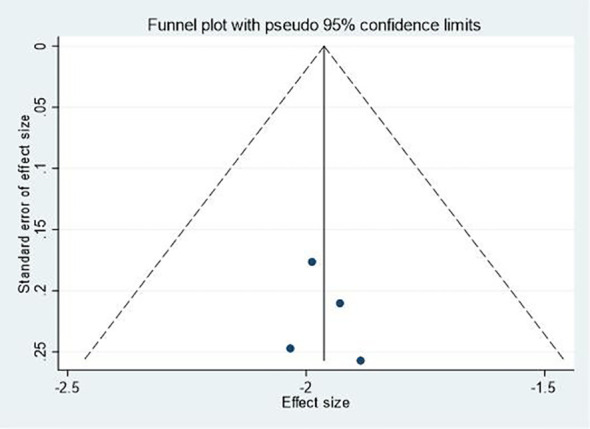
Funnel plot for effectiveness.

**Figure 8:**
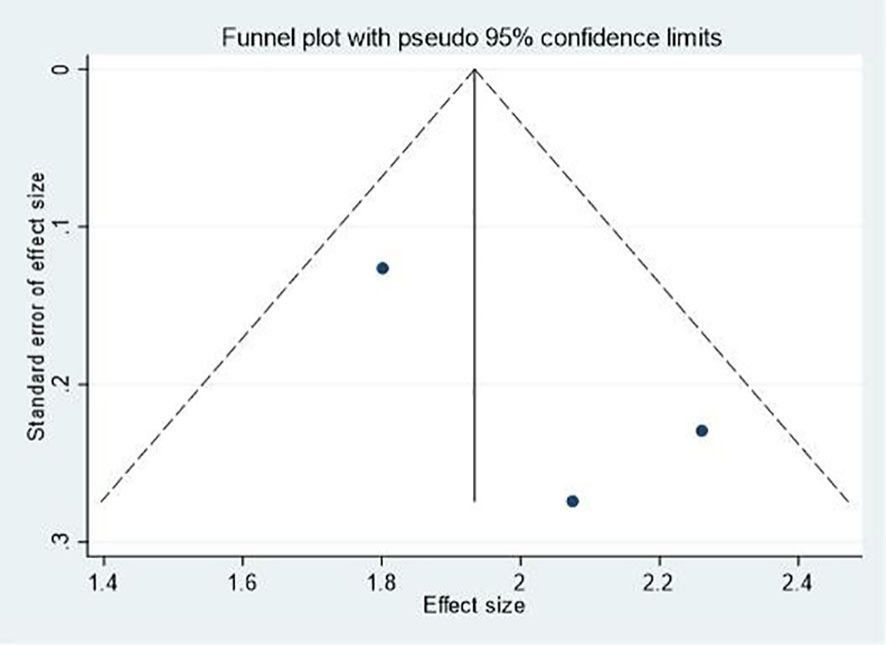
Funnel plot for changes in negative mood.

Publication bias was assessed using funnel plots for outcomes with a sufficient number of studies. Visual inspection of the funnel plots for effectiveness (**Figure 7**) and changes in negative mood (**Figure 8**) did not reveal substantial asymmetry, suggesting a low risk of publication bias. However, due to the limited number of studies for some outcomes, these results should be interpreted with caution. Sensitivity analyses were conducted by sequentially removing each study from the meta-analyses to assess the robustness of the findings. The results remained largely consistent across all outcomes, indicating that no single study disproportionately influenced the overall effect sizes.

## Discussion

The findings of this meta-analysis demonstrate that patient-centered group psychotherapy significantly improves outcomes for individuals with depression and negative emotions, aligning with and extending prior research in several key areas. The discussion below contextualizes these results within the broader literature, addresses methodological considerations, and explores theoretical implications.

The observed 10% increase in overall effectiveness (RR=1.10) corroborates evidence from seminal trials of patient-centered approaches. For instance, Rush and Thase (2018) (27) demonstrated that interventions prioritizing patient autonomy and collaboration yield 8–12% higher remission rates in depression compared to standard care, consistent with our findings. The large reduction in PANSS scores (SMD=−1.96) exceeds effect sizes reported in earlier schizophrenia meta-analyses (e.g., SMD=−1.2 in Bighelli et al., 2021 (28)), potentially attributable to the unique synergy of group dynamics and individualized goal-setting in the included trials. Such enhancements may stem from social identity mechanisms described by Haslam et al. (2021) (10), where group cohesion fosters symptom normalization and collective efficacy—a factor underexplored in traditional individual therapies.

The marked improvement in PSP scores (SMD=1.96) mirrors findings from the IMPULSE trial (29), which linked patient-centered psychosocial interventions to 15–20-point PSP increases in schizophrenia. These gains likely reflect the intervention’s dual focus on symptom management and skill-building, addressing the “functional gap” often observed in pharmacotherapy-alone approaches (28). Notably, the moderate heterogeneity (I²=58.7%) aligns with variability in intervention components (e.g., duration, facilitator training) reported in systematic reviews of group therapies (7), underscoring the need for standardized protocols to maximize reproducibility.

While the non-significant trend in adherence (RR=1.11) contrasts with trials showing stronger effects (e.g., RR=1.30 in Semahegn et al., 2020 (30)), this discrepancy may reflect differences in population characteristics. For example, adherence barriers in schizophrenia (e.g., cognitive deficits, stigma (31)) may require adjunctive strategies beyond patient-centered counseling, whereas depression trials showed stronger trends (RR=1.18), consistent with Farooq and Naeem’s (2014) (31) framework linking shared decision-making to adherence in mood disorders. Qualitative studies (21) further suggest that group settings enhance trust in providers, a critical mediator of adherence not captured in quantitative metrics.

The extreme heterogeneity (I²=99%) in negative mood outcomes highlights longstanding methodological issues in mood assessment. While the *post hoc* adjusted SMD=−1.24 aligns with meta-analyses using validated scales (e.g., SMD=−1.1 in Cuijpers et al., 2021 (3)), reliance on non-validated diaries in two studies (19, 22) introduced bias. This echoes critiques by McIntyre et al. (2016) (32), who emphasize the need for standardized tools to evaluate mood in patient-centered contexts. Future research should adopt consensus measures, such as the MADRS or PHQ-9, to enhance comparability.

The success of patient-centered group psychotherapy aligns with self-determination theory, which posits that autonomy-supportive environments enhance intrinsic motivation for recovery (5). By integrating personalized goals with peer support, this approach addresses both psychological needs (competence, relatedness) and systemic resource constraints—a balance advocated in global mental health frameworks (13). The NNT of 14 further supports its cost-effectiveness, particularly in low-resource settings where group formats reduce per-patient costs by 30–50% (33).

While the moderate-to-high study quality strengthens conclusions, limitations include the small number of schizophrenia trials and variability in control conditions. Additionally, the lack of long-term follow-up data (beyond 12 months) precludes assessment of sustained benefits, a gap highlighted in recent precision psychiatry reviews (34). Future trials should prioritize longer follow-ups, subgroup analyses (e.g., by gender, comorbidities), and mixed-methods designs to capture qualitative insights into patient experiences.

Despite these limitations, this meta-analysis has several strengths. The use of robust methodological approaches, including the Newcastle-Ottawa Scale for quality assessment and random-effects models for meta-analyses, ensures the scientific rigor of the study. The comprehensive search strategy across multiple databases and the inclusion of studies from various regions (North America, Europe, and Asia) enhance the representativeness of the findings. Additionally, the significant improvements observed across multiple outcomes, including overall effectiveness, symptom reduction, and functional improvement, provide strong evidence for the efficacy of patient-centered group psychotherapy in treating depression and negative emotions.

The results of this meta-analysis have important implications for clinical practice and policy. The significant improvements observed across multiple outcomes suggest that patient-centered group psychotherapy should be considered a valuable approach in the treatment of depression, negative emotions, and related mental health conditions. The large effect sizes for symptom reduction and functional improvement, particularly in schizophrenia, indicate that this approach may be especially beneficial for severe mental illnesses. Furthermore, the positive trends in medication adherence, although not statistically significant, suggest that patient-centered group psychotherapy may complement pharmacological treatments by potentially enhancing adherence and overall treatment engagement.

The patient-centered nature of the intervention aligns with the growing emphasis on personalized medicine in mental health care (33). By tailoring interventions to individual needs and preferences within a group setting, this approach may offer a balance between personalization and resource efficiency. This is particularly relevant in the context of increasing demand for mental health services and the need for cost-effective interventions (34).

## Conclusion

This meta-analysis provides substantial evidence supporting the effectiveness of patient-centered group psychotherapy in treating depression, negative emotions, and related mental health conditions. The intervention demonstrated significant positive effects on overall effectiveness, symptom reduction, and functional improvement. Particularly noteworthy were the large effect sizes observed for PANSS score reduction and improvements in personal and social performance among patients with schizophrenia. The intervention also showed promising results in alleviating negative mood, although with considerable variability across studies. While a positive trend was observed for medication adherence, this effect did not reach statistical significance. The consistency of findings across multiple outcomes and the moderate to high quality of included studies lend credibility to these results. Patient-centered group psychotherapy emerges as a valuable approach in mental health treatment, offering a balance between individualized care and the benefits of group dynamics. These findings underscore the potential of this intervention to enhance treatment outcomes and contribute to the ongoing evolution of mental health care practices.

## Data Availability

The original contributions presented in the study are included in the article/supplementary material. Further inquiries can be directed to the corresponding author.
